# Performance and Durability of Biopolymer Blends Containing Modified Metal Oxide Particles

**DOI:** 10.3390/polym17223000

**Published:** 2025-11-11

**Authors:** Giulia Infurna, Andrea Antonino Scamporrino, Elisabetta Morici, Elena Bruno, Giuseppe Pecoraro, Nadka Tz. Dintcheva

**Affiliations:** 1 Dipartimento di Ingegneria, Università di Palermo, Viale delle Scienze, Ed. 6, 90128 Palermo, Italy; giulia.infurna@unipa.it (G.I.); elisabetta.morici@unipa.it (E.M.); giuseppe.pecoraro09@community.unipa.it (G.P.); 2 Institute for Polymers, Composites, and Biomaterials CNR-IPCB, Via P. Gaifami 18, 95126 Catania, Italy; andreaantonio.scamporrino@cnr.it; 3 ATEN Center, Università di Palermo, Viale delle Scienze, Ed. 18, 90128 Palermo, Italy; 4 Department of Physics and Astronomy “E. Majorana”, Institute for Micro-Electronics and Microsystems IMM-CNR, University of Catania, Via S. Sofia 64, 95123 Catania, Italy; elena.bruno@dfa.unict.it

**Keywords:** biopolymer blends, modified metal oxides, solid state properties, morphology refinement, photo-oxidation resistance

## Abstract

This study applies circular and sustainable principles to the formulation of biopolymer-based materials using naturally occurring additives. To improve the affinity between the host matrix and additives such as metal oxides, the work involves adding stearic acid-modified zinc oxide (*f*-ZnO) and sonicated titanium dioxide (*s*-TiO_2_) to a polylactic acid and bio-derived polyamide 11 (PLA/PA11 = 70/30 *w*/*w* biopolymer blend via melt mixing. To evaluate the impact of the functionalization and sonication on metal oxides (i.e., *f*-ZnO and *s*-TiO_2_) introduced into the PLA/PA11 blend, composites containing unmodified ZnO and TiO_2_ prepared under the same processing conditions were compared with the modified ones. All of the composites were characterised in terms of their solid-state properties, morphology, melt behaviour, and photo-oxidation resistance. The addition of both *f*-ZnO and *s*-TiO_2_ appears to exert a plasticising effect on the rheological behaviour, in contrast to unmodified ZnO and TiO_2_. The presence of stearic acid tails on ZnO has been estimated at approximately 4%, whereas sonication reduces the diameter of TiO_2_ particles by half. In the solid state, both unmodified and modified particles can reinforce the biopolymer matrix, enhancing the Young′s (elastic) modulus. Calorimetry analysis suggests that unmodified and modified metal oxide particles do not influence the glass transition of the PLA phase but affect the melt temperatures of both biopolymeric phases by reducing macromolecular mobility. Morphology analysis shows that the presence of both f-ZnO and s-TiO_2_ particles does not reduce the size of the PA11 droplets. The f-ZnO particles, which have long stearic tails and are more compatible with the less-polar phase (PLA), are probably located at the interface between the two biopolymeric phases or in the PLA phase. Furthermore, *s*-TiO_2_ particles, like TiO_2_, do not reduce the dimensions of PA11 droplets, suggesting that there is no preferential location of the particles. Due to the presence of both *f*-ZnO and *s*-TiO_2_, an increase in the hydrophobicity of the PLA/PA11 blend has been detected, suggesting enhanced water resistance. The photo-oxidation resistance of the PLA/PA11 blend is significantly reduced by the presence of unmodified metal oxides and even more so by the presence of modified metal oxides. This suggests that metal oxides could be considered photo-sensitive degradant agents for biopolymer blends.

## 1. Introduction

The integration of biopolymers and bioplastics contributes significantly to the advancement of sustainability objectives by minimising dependence on petrochemical-derived plastics, facilitating the development of materials with partial or complete compostability, and supporting the transition toward a circular economy model. Nonetheless, the intrinsic limitations of certain individual biopolymers—such as suboptimal mechanical, thermal, or barrier properties—may restrict their applicability in high-performance domains. To overcome these constraints, the formulation of biopolymer-based blends presents a strategic approach to tailor material properties for specific end-use requirements. These engineered blends can be employed across a wide range of sectors, including but not limited to packaging, biomedical devices, agriculture, and consumer goods [1,2,3,4,5].

Single biopolymers often exhibit inherent limitations, including poor thermal stability, brittleness, and slow biodegradation rates. To overcome these drawbacks, blending strategies can be employed to enhance mechanical strength, barrier performance, thermal resistance, and overall processability. For example, polylactic acid (PLA), a widely used biopolymer, is known for its brittleness and limited impact resistance. However, its properties can be significantly improved by blending with other biopolymers such as polybutylene succinate (PBS) or polyhydroxyalkanoates (PHAs), resulting in increased flexibility and toughness. Alternative blending strategies include combining polylactic acid (PLA) with starch-based biopolymers to improve biodegradability [6,7,8], or blending PLA with polyamide 11 to tailor morphology and optimise solid-state properties [9,10,11]. Our previous studies indicate that a 70/30 *w*/*w* ratio of PLA to PA11 provides an optimal balance, effectively modulating morphology while enhancing the solid-state properties of the blend [12,13,14]. Furthermore, blending biopolymers with other biopolymers can serve to reduce costs, such as by incorporating waste-derived biopolymers from cellulose or chitin, or to tailor functionality through the use of biopolymers bearing specific functional groups [15,16,17,18]. Additionally, investigating the behaviour of compatible or incompatible biopolymer blends is important when considering items that are collected together and in some cases cannot be economically separated at the end of their life.

However, strategies for customising the functional properties and performance of biopolymers or biopolymer blends involve adding naturally occurring particles, such as clay, minerals, metal oxides, colourants, hydrophobic/hydrophilic additives, and antimicrobial, anti-bacterial, and anti-oxidant additives [19,20]. Many of these additives must be added in the molten state of the biopolymers, and achieving their effective dispersion and distribution can be challenging. Therefore, the introduction of coupling agents, compatibilisers, and dispersants can successfully improve the dispersion and distribution of these additives. Therefore, the chemical and/or physical modification of the additives could reduce the need for further additives.

Numerous studies have shown that the incorporation of metal oxide nanoparticles—such as zinc oxide (ZnO), titanium dioxide (TiO_2_), copper oxide (CuO), tin dioxide (SnO_2_), and silver oxide (Ag_2_O)—into polymer matrices can significantly enhance their physicochemical properties. These enhancements include increased mechanical strength, thermal stability, antimicrobial activity, electrical conductivity, optical performance, and surface functionality. As a result, polymer nanocomposites reinforced with metal oxides have been successfully applied in diverse fields such as sensor technology, photocatalysis, water purification, membrane separation, ion-exchange systems, and adsorption processes. Additionally, metal oxides can fine-tune the optical, structural, electrical, and anti-bacterial characteristics of both conventional and bio-based polymers, making them promising materials for high-performance and sustainable packaging solutions [21,22,23,24].

Zinc oxide (ZnO) and titanium dioxide (TiO_2_) are among the most widely utilised metal oxide fillers in the fabrication of polymer nanocomposites. ZnO, in particular, is a cost-effective and chemically accessible material characterised by its high redox potential. These properties enable it to effectively degrade organic pollutants and exhibit strong antimicrobial activity, making it valuable for applications requiring both environmental remediation and microbial control [25,26]. Titanium dioxide (TiO_2_) is characterised by its structural stability, non-toxicity, corrosion resistance, and photocatalytic properties. Upon excitation by UV light, both ZnO and TiO_2_ exhibit photocatalytic activity through the generation of electron–hole pairs (e^−^ and h^+^). These charge carriers react to form hydroxyl radicals and reactive oxygen species (ROS), which disrupt the metabolic processes of pollutants and microorganisms, thereby inhibiting their growth or inducing cell death [27,28,29].

Although introducing metal oxide particles can be an advantageous method of improving certain macroscopic properties, it is sometimes necessary to modify these particles to prevent agglomeration, improve dispersion during melt processing, and enhance interfacial bonding with the polymer matrix. One industrially applicable and inexpensive method of modifying ZnO is to introduce stearic acid tails. The stearic acid binds to the ZnO through the -COOH groups while the hydrophobic tail remains free and can be exposed to the polymer matrix, thereby improving dispersion and enhancing bonding with the host matrix. Considering an industrially viable and inexpensive method of modifying TiO_2_ particles, ultrasonication can be proposed as a suitable method. This treatment prevents agglomeration, assists dispersion in liquids or polymer matrices, enhances surface area and reactivity, and tunes the optical and photocatalytic properties [30,31].

Based on our previous experience, the optimal blend of polylactic acid (PLA)/polyamide (PA11) is 70/30 *w*/*w* [13]. Incorporating zinc oxide (ZnO) and titanium dioxide (TiO_2_) at a low concentration of 0.5 wt.% can modify the final properties and blend morphology profitably [12,14]. Based on this, the current study examines the incorporation of modified metal oxide particles, such as stearic acid-functionalised ZnO (*f*-ZnO) at different concentrations of 0.5, 1, and 2 wt.% as well as ultrasound-treated titanium dioxide (*s*-TiO_2_) at 1 wt.%, into a PLA/PA11 = 70/30 *w*/*w* blend. This work focuses on modifying the blend morphology to modify the selective localisation and dispersion of the metal oxides, thereby enhancing the solid-state properties and surface hydrophobicity. The study of incompatible polymer or biopolymer blends is important, particularly when considering the potential for recycling and upcycling these blends.

Modifying the ZnO and TiO_2_ through chemical functionalisation and ultrasound treatment, respectively, is also considered to reduce the use of blend constituents. For comparison purposes, a PLA/PA11 = 70/30 *w*/*w* and the blends containing unmodified ZnO and TiO_2_ particles were also produced by melt mixing under the same processing conditions. All the results obtained highlight that modifying metal oxides leads to the formulation of particles with improved performance, which can refine the blend morphology and enhance its solid-state properties and performance.

## 2. Materials and Methods

### 2.1. Materials

The polylactic acid (PLA) used in this work was of commercial extrusion sheet grade, supplied by NatureWorks (Blair, NE, USA, named PLA 2002D), with an average number molecular weight of about 121,000 g/mol, a ratio of 96% L-lactide to 4% D-lactide units, and a melt flow index 6 g/10 min (230 °C, 2.16 kg).

The polyamide used in this work was a Polyamide 11, PA11, (Nylon 11, pellet form, from Sigma Aldrich, St. Louis, MO, USA), with glass transition temperature Tg = 46 °C, melting temperature Tm = 198 °C, and density ρ = 1.026 g/cm3 at 25 °C, molecular weight Mw = 201.31 g/mol, and MFI@235 °C/2.16 kg = 14.5 ± 1.2 g/10 min.

Zinc oxide (ZnO) nanopowder (<100 nm particle size) was purchased from Sigma Aldrich and used without any further purification. According to our previous investigations, ZnO nanoparticle size spanned a 100–200 nm range, with a mix of tubular and round-shaped forms [12].

Titanium dioxide (TiO_2_, AEROXIDE^®^ TiO_2_ P25) was purchased from Evonik (Essen, Germany) and used without further purification. Its features are specific surface area (BET): 35–65 m^2^/g; and tamped density: approximately 140 g/L, loss on drying < 1.5%, pH-value 3.5–4.5, and SiO_2_ content < 0.2%.

Stearic acid (Octadecanoic acid, chemical formula: CH_3_(CH_2_)_16_COOH) was purchased from Sigma-Aldrich (St. Louis, MO, USA) and used without further purification. It is a reagent-grade sample with a purity of 95%, supplied in powder form. The stearic acid main physical characteristics are: melting point: ~69–70 °C; boiling point (decomposes): ~383 °C at 760 mmHg; and decomposition onset: ~360 °C.

### 2.2. Modification of Metal Oxides

#### 2.2.1. Modification of ZnO to f-ZnO

Six grams (0.07372 mol) of ZnO were dispersed in 200 mL of a 1:2 (*v*/*v*) water/ethanol mixture and subjected to ultrasonication for 20 min. Subsequently, 8.2 g (0.20501 mol) of NaOH pellets were added to the dispersion, and the mixture was sonicated until complete dissolution of the solid. Simultaneously, 0.6 g (0.00211 mol) of stearic acid was added to 100 mL of a 1:2 (*v*/*v*) water/ethanol mixture and sonicated for 15 min. The two resulting solutions were then combined and further sonicated for 40 min. The final mixture was divided into four vessels and centrifuged at 4500 rpm for 10 min using a CL10 centrifuge (Thermo-Scientific). The supernatant in each vessel was discarded and replaced with ethyl acetate. The vessels were then sonicated for 5 min to redisperse the sedimented solid and enhance washing efficiency. This centrifugation/washing step was repeated three times at 3500 rpm for 10 min per cycle to remove all unbound stearic acid from the ZnO surface. After the final centrifugation step, the collected solid was dried using a rotavapor and subsequently placed in a vacuum oven at 60 °C overnight.

#### 2.2.2. Sonication of TiO_2_ to s-TiO_2_

An aqueous suspension of titanium dioxide particles using deionised water was subjected to ultrasonication treatment for 2 h; then, *s*-TiO_2_ particles were subjected to vacuum drying at 80 °C overnight.

### 2.3. Melt Blending

Both biopolymers PLA and PA11 and the metal oxides were vacuum-dried at 80 °C overnight before processing to avoid hydrolytic degradation during melt processing. Blend PLA/PA11 = 70/30 *w*/*w* (named PLA70), and PLA/PA11 containing unmodified and modified metal oxides at varying amounts were prepared by melt mixing using a Brabender PLA-330 internal mixer at 200 °C for 5 min at 50 rpm. Both ZnO and *f*-ZnO were been added at 0.5, 1, and 2 wt.%, whereas both TiO_2_ and *s*-TiO_2_ were been introduced at 1 wt.% in PLA/PA11 = 70/30 *w*/*w*. To improve the ZnO, *f*-ZnO, TiO_2_, and *s*-TiO_2_ dispersion in PLA/PA11 blend melt, the biopolymers were pre-mixed for 1 min and then the metal oxides were added: the mixing continued for another 4 min.

Thin films, having a thickness of ca. 100 microns, requested for the characterisations were obtained by the hot compression moulding process.

### 2.4. Characterisations of Particles

#### 2.4.1. ATR-FTIR Spectroscopy

To examine the chemical structures attenuated total reflectance Fourier-transform infrared (ATR-FTIR) spectroscopy was performed using a Perkin-Elmer Spotlight 200i FTIR Microscopy System (710 Bridgeport Avenue, Shelton, CT, USA) with spectrum 3. A wavelength ranging from 600 to 4000 cm^−1^ was used at a resolution of 4 cm^−1^, with 32 scans.

#### 2.4.2. TGA/DTG Analysis

Thermogravimetric analysis (TGA) was conducted in a Netzsch Jupiter F1 STA 449 (NETZSCH B.V & Co. Holding KG, Selb, Germany) in the range 30–900 °C, with a heating rate of 10 °C min^−1^, by flushing 20 mL min^−1^ of nitrogen during the analysis.

#### 2.4.3. SEM and TEM Analysis

The particles were observed by scanning electron microscopy images (SEM, Gemini 152 field emission SEM Supra 25, Carl Zeiss, Oberkochen, Germany).

Transmission electron microscopy (TEM) observations were carried out using a Jeol JEM-2100 (Musashino, Akishima-shi, Tokyo, Japan) at 200 kV.

### 2.5. Characterisations of Composites

#### 2.5.1. FTIR Spectroscopy

A Fourier-transform infrared spectrometer (Spectrum One, Perkin Elmer, Shelton, CT, USA) was used to record IR spectra using 16 scans at a resolution of 1 cm^−1^. The progress of photo-oxidation degradation of the samples has been followed by FTIR analysis, monitoring the variations of carbonyl range (1850–1600 cm^−1^) in time using Spectrum 10.4.2.279 software.

#### 2.5.2. Water Contact Angle (WCA)

The water contact angle (WCA) was measured at room temperature using a First Ten Angstrom (Portsmouth, VA, USA) FTA1000C system (Data Physics Instruments, Filderstadt, Germany), with demineralized water. The films were fixed on top of a plane solid support and kept flat during water deposition and acquisition. The sessile drop method was used with a droplet volume of 6 μL.

#### 2.5.3. Tensile Tests

Tensile tests were carried out using a universal testing machine (Instron model 3365, High Wycombe, UK), according to the ASTM D882 method [32], on rectangular samples. The tests were performed using a tensile speed of 1 mm/min for 1 min to evaluate the Young’s modulus, and then the velocity was increased to 10 mm/min until sample breakage. The tensile test has been performed on ten samples of each material.

#### 2.5.4. Rheological Analysis

Rheological tests were performed using a stress-controlled rheometer (ARES G-2) in parallel plate geometry (plate diameter 25 mm). The complex viscosity (η*), storage (G′), and loss (G″) moduli were measured under frequency scans from ω = 0.1 to 100 rad/s at T = 170 °C. The strain amplitude was γ = 5%, which preliminary strain sweep experiments proved to be low enough to be in the linear viscoelastic regime.

#### 2.5.5. Scanning Electron Microscopy (SEM)

The morphologies of the blend nanocomposites were investigated by scanning electron microscopy images (SEM, Gemini 152 field emission SEM Supra 25, Carl Zeiss, Oberkochen, Germany). The images were obtained in the Inlens mode at 5 kV. The samples of neat PLA and PLA/PA11 were cryogenically fractured in liquid nitrogen to obtain a cross-section and subsequently sputter-coated with gold (10 mA, 4 min) to create a conductive surface layer of 10 nm.

The average numerical diameter (*d_n_*), Equation (1), average volumetric diameter (*d_v_*), Equation (2), dispersion (*D*), Equation (3), and the diameters of the dispersed phase were determined by measurements on the obtained SEM images, containing more than one hundred dispersed phase particles (*n_i_* PA11 particles, having *d_i_* diameters) per sample, were calculated according to the following equations:

(1) $$d_{n}=\frac{\Sigma_{i}n_{i}d_{i}}{\Sigma_{i}n_{i}}$$

(2) $$d_{v}=\frac{\Sigma_{i}n_{i}d_{i}^{4}}{\Sigma_{i}n_{i}d_{i}^{3}}$$

(3) $$D=\frac{d_{v}}{d_{n}}$$

For particles having ellipse form, an equivalent diameter, Equation (4), was used through the particle area, according to the following equation:

(4) $$d_{eq}=2{\left(\frac{A}{\pi }\right)}^{\frac{1}{2}}$$

where *π* = 3.14 and *A* is the surface.

#### 2.5.6. Differential Scanning Calorimetry (DSC)

The calorimetric analysis was evaluated by differential scanning calorimetry (DSC), using a TA Instruments Q100 in a nitrogen atmosphere on 5 ± 0.5 mg samples sealed in aluminium crucibles. Samples were heated from ambient temperature to 230 °C at 10 °C/min, and held in equilibrium conditions for 3 min at 230 °C to eliminate the thermal history, cooled to 25 °C at 10 °C/min, and then reheated to 230 °C at 10 °C/min.

### 2.6. Photo-Oxidation Exposure

Accelerated photo-oxidation was carried out using a Q-UV/basic weatherometer (from Q-LAB, Westlake, OH, USA) equipped with UVB lamps (313 nm). The weathering conditions consisted of continuous light irradiation at T = 70 °C.

The progress of the photo-oxidation of PLA/PA11 = 70/30 *w*/*w* and blends containing metal oxides was monitored by FTIR analysis at different exposure times. The height of the peak at 1845 cm^−1^ was assigned to the accumulation of the anhydride functionalities in the PLA phase due to PLA photo-oxidation, and the height of the peak at 1725 cm^−1^ was assigned to the accumulation of carbonyl functions in the PA11 phase due to PA11 photo-oxidation [14]. The spectra were not normalised because it is impossible to correctly identify an appropriate peak for normalisation in the blend systems.

## 3. Results and Discussion

### 3.1. Characterisations of Biopolymer Blend Containing f-ZnO

**Figure 1:**
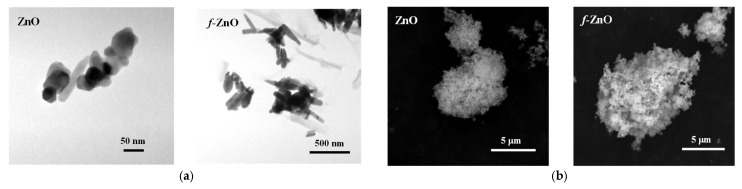

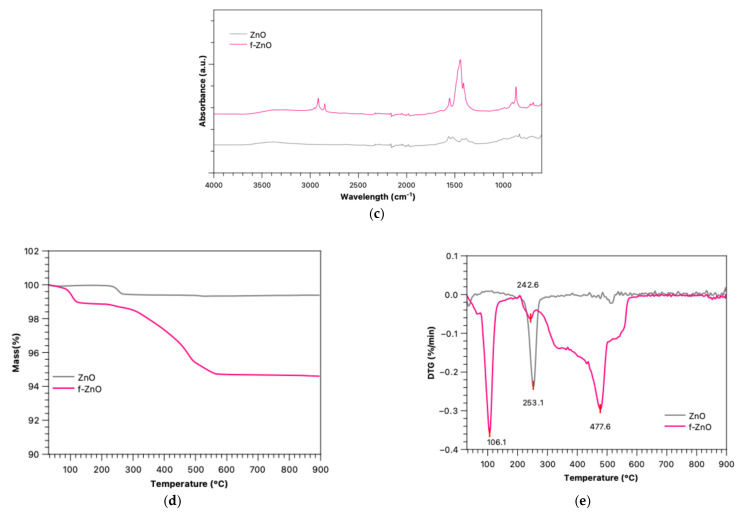
(**a**) TEM images (**left**), (**b**) SEM images (**right**), (**c**) ATR-FTIR spectra, (**d**) TGA curves, and (**e**) DTG trends of ZnO and *f*-ZnO.

To investigate the effect of modifying the surface of ZnO to form *f*-ZnO through stearic acid functionalisation, TEM and SEM observations and ATR-FTIR and TGA analyses of the particles were carried out. Figure 1a,b show TEM and SEM images of ZnO and *f*-ZnO particles before their incorporation into the biopolymer blend via melt mixing. The *f*-ZnO particles exhibit an elongated morphology and are larger in size when compared to unmodified ZnO particles. Overall, the surface of the f-ZnO particles appears rougher, which is likely due to the presence of the stearic acid modifier. Although the images show slight differences between ZnO and *f*-ZnO, the particle sizes cannot be estimated exactly as clusters are visible rather than individual particles. Figure 1c shows the ATR_FTIR spectra of ZnO and *f*-ZnO. The spectrum of ZnO does not show any visible peaks, and there are small shoulders at >1600 cm^−1^ due to the intrinsic structure of ZnO. The spectrum of f-ZnO shows well-visible peaks that can be assigned to the presence of stearic acid tails. According to the literature [33], stearic acid shows well-visible peaks at 2922 and 2851 cm^−1^ due to the symmetric and asymmetric stretching of -CH groups, and the peak at 1705 cm^−1^ is assigned to the symmetric stretching of -COOH groups. In the spectrum of *f*-ZnO are visible small peaks at 2918 and 2850 cm^−1^, which are assigned to the symmetric and asymmetric stretching of -CH groups, and at 1445 cm^−1^, which is assigned to the bending of -CH. The peaks at 1553 and 1404 cm^−1^ are due to the symmetric and asymmetric stretching of -COO^-^ groups. The latter confirms unequivocally the presence of steric acid tails on ZnO particles. Further confirmations come from TGA/DTG analysis of ZnO and *f*-ZnO, see Figure 1d,e. The ZnO shows a small mass loss (less than 1%) at ca. 253.1 °C due to the reorganisation of its structure upon temperature increase. The *f*-ZnO exhibits a mass loss of 1% at 106.1 °C and 242.6 °C (total at both temperatures), probably due to the presence of a small amount of humidity and ZnO reorganisation, respectively. The well-pronounced mass loss at ca. 477.6 °C can be attributed to the stearic tails’ decomposition (ca. 5%), suggesting the formation of Zn stearate, which is in agreement with the literature regarding the decomposition of calcium stearate which occurs at temperatures significantly higher than the temperature of decomposition of stearic acid (approximately 250–300 °C) [34]. This again confirms the successful functionalisation of ZnO to f-ZnO with the presence of stearic acid tails at ca. 4% on the ZnO particles.

Given the excellent intrinsic properties of ZnO, its relatively low cost, and its potential for large-scale industrial applications, both unmodified ZnO and modified f-ZnO were introduced at three different concentrations (0.5, 1, and 2 wt.%) in a 70/30 *w*/*w* PLA/PA11 blend via melt mixing.

**Figure 2:**
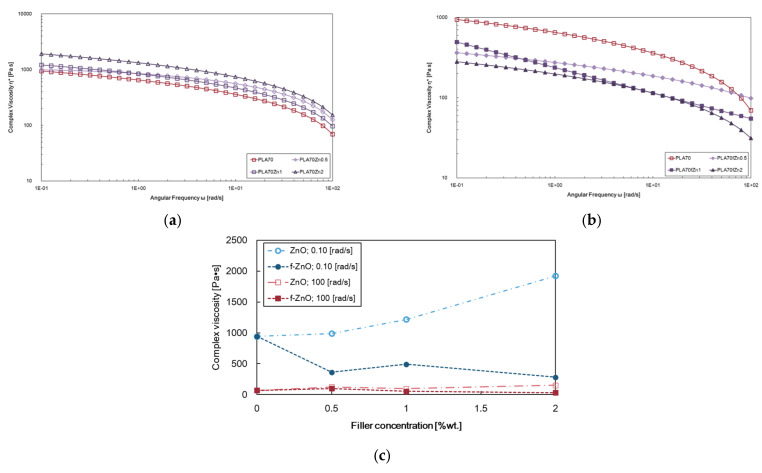
Complex viscosity curves of PLA/PA11 = 70/30 *w*/*w* (named “PLA70” in the figure) and materials containing (**a**) ZnO and (**b**) *f*-ZnO at different concentrations as a function of frequency; (**c**) complex viscosity values at 0.1 rad/s and 100 rad/s.

Figure 2a,b show the viscosity curves of PLA/PA11 = 70/30 *w*/*w* containing ZnO and *f*-ZnO at different amounts, i.e., 0.5, 1, and 2 wt.%, while Figure 2c displays the complex viscosity value trends at low (0.1 rad/s) and high (100 rad/s) frequencies as a function of filler content. The biopolymer blend, i.e., PLA/PA11 = 70/30 *w*/*w*, exhibits less-pronounced Newtonian behaviours at low frequencies and pronounced shear thinning at high frequencies. Furthermore, the biopolymer blend exhibits a gradual decrease in complex viscosity due to the incompatibility of the biopolymer constituents and the macromolecular orientation in the direction of the oscillatory test. The presence of unmodified ZnO leads to an increase in viscosity, with all curves appearing parallel at values higher than those of the PLA/PA11 = 70/30 *w*/*w* curve (named “PLA70” in the figure). Unmodified ZnO does not alter the rheological behaviour of the blend matrix and can exert a pronounced reinforcement effect, see Figure 2a. In contrast, the presence of *f*-ZnO significantly reduces viscosity values, suggesting a slight change in the overall rheological behaviour, see Figure 2b. As can be seen, the slopes of the viscosity curves for samples containing different amounts of *f*-ZnO are different. More specifically, shear thinning is less pronounced up to the point of disappearance for samples containing a high amount of *f*-ZnO. Changes in rheological behaviour can be explained by considering the pronounced sleeping effect of the stearic acid modifier on the macromolecules of the blend matrix. The presence of the modifier favours movement and orientation of the macromolecules during the oscillatory test, leading to a reduction in viscosity. Similar considerations can be made considering the trends of both G′ and G″ moduli, reported as Figures S1 and S2 in the Supplementary Information.

However, the rheological behaviour of these blends is mainly governed by the PLA phase, although all the blends exhibit shear thinning in the high-frequency range (e.g., 1–100 rad/s) and this is well pronounced for *f*-ZnO.

**Figure 3:**
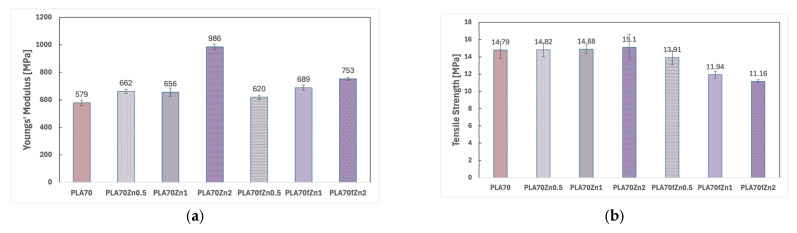

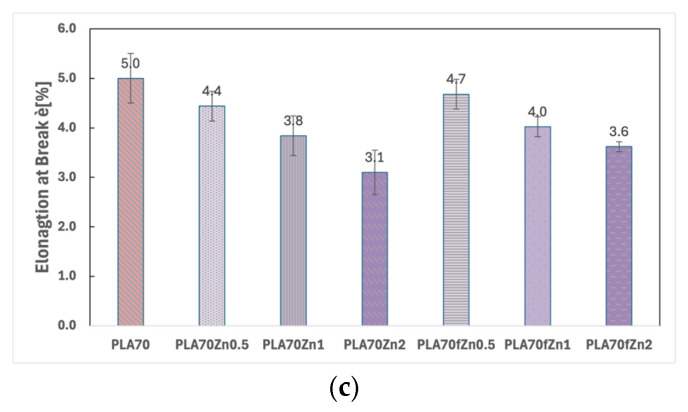
Main mechanical properties: (**a**) Young’s modulus, E, (**b**) tensile strength, TS, and (**c**) elongation at break, EB, of PLA/PA11 = 70/30 *w*/*w* (named “PLA70” in the figure) and materials containing ZnO and *f*-ZnO at 0.5, 1, and 2 wt.%.

Mono-axial tensile tests were performed to evaluate the mechanical strength of the produced sample. Figure 3a–c show the main mechanical properties (Young’s modulus (E), tensile strength (TS), and elongation at break (EB)) of all the biopolymer blends investigated, which contain both unmodified and modified ZnO. Young’s modulus values increase with the addition of both unmodified and modified ZnO as filler content increases. The presence of unmodified ZnO appears to lead to greater rigidity enhancement. In contrast, the presence of modified ZnO increases rigidity but exerts a reduced reinforcing effect in the solid state, probably due to the plasticising effect of the stearic acid modifier. The elongation at break values of samples containing both ZnO and f-ZnO are slightly lower than the EB value of the biopolymer blend. Therefore, the biopolymer blend exhibits brittle behaviour, as well as the samples containing both unmodified and modified ZnO. The presence of ZnO does not affect the tensile strength, whereas f-ZnO causes a slight decrease due to its plasticising effect.

Therefore, the PLA/PA11 exhibits brittle behaviour due to incompatibility between the two constituents. As expected, the addition of unmodified and modified solid particles slightly exacerbated the brittle behaviour of the blend.

**Figure 4:**
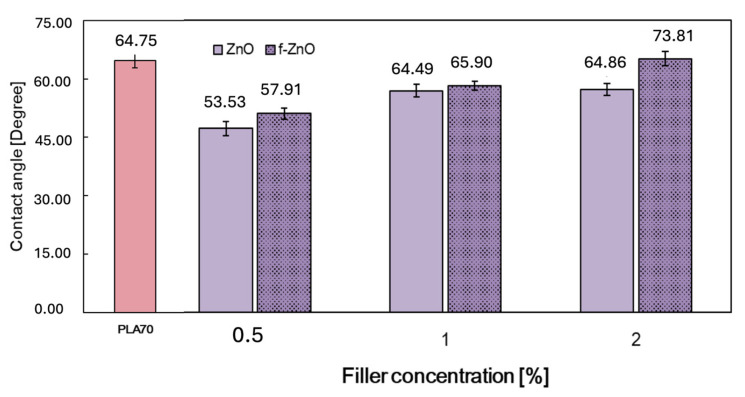
Water contact angle (WCA) measurements of PLA/PA11 = 70/30 *w*/*w* (named “PLA70” in the figure) and materials containing ZnO and *f*-ZnO at 0.5, 1, and 2 wt.%.

Water contact angle (WCA) measurements were performed to investigate the hydrophobicity of the films’ surfaces, specifically examining how this property changes upon the addition of ZnO and *f*-ZnO at varying concentrations. Figure 4 shows the WCA values of the biopolymer blend matrix and materials containing both ZnO and *f*-ZnO at different concentrations. The presence of both unmodified and modified ZnO in small amounts (0.5 wt.%) appears to decrease WCA values. The presence of 1 wt.% of both ZnO and *f*-ZnO does not affect hydrophobicity. However, at 2 wt.%, it appears that the WCA increases for *f*-ZnO, thereby enhancing surface hydrophobicity.

**Figure 5:**
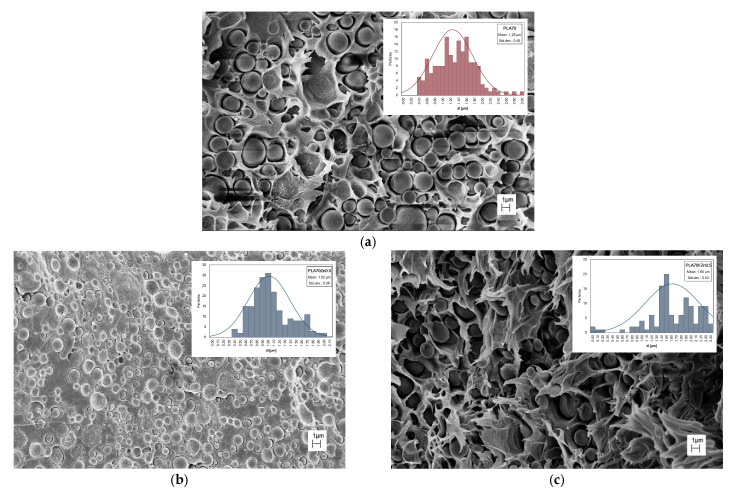
SEM observations and the inserts show the distribution of PA11 particle dimensions of (**a**) PLA/PA11 = 70/30 *w*/*w* and materials containing (**b**) ZnO at 0.5 wt.% and (**c**) *f*-ZnO at 0.5 wt.%.

Morphology observations were performed using scanning electron microscopy (SEM). Figure 5a–c show representative SEM images of the PLA/PA11 = 70/30 *w*/*w* blend and samples containing 0.5 wt.% ZnO and *f*-ZnO. The inserts in Figure 5a–c show the distribution of PA11 particle dimensions. Table 1 reports the average diameter (*d_n_*) of PA11 particles in PLA and the ratio (*D*) between *d_v_* and *d_n_* in PLA/PA11. According to previous studies, the PLA/PA11 = 70/30 *w*/*w* blend exhibits a droplet morphology, with the two biopolymer phases clearly distinguishable. More specifically, the droplet matrix morphology shows a continuous PLA phase and a roughly spherical PA11 dispersed phase. The presence of ZnO particles appears to significantly reduce the dimensions of the PA11 particles, while *f*-ZnO causes a slight increase. This is consistent with the appearance of the particle dimensions before blending with the biopolymer matrix.

According to previous studies [13,14], clay and metal oxide particles are primarily disposed in the interphase between the two biopolymer phases, with only a small amount placed in the more polar polymeric phase, e.g., PA11, at low concentrations: this reduces the dimensions of the droplet-dispersed phase. The current study confirms this for unmodified ZnO particles, see Table 1. The situation is more complicated for *f*-ZnO, particularly given that the stearic tails are more compatible with the less-polar phase, e.g., PLA, and this makes the PA11 particles appear larger. It is worth noting that the morphology of the blend containing f-ZnO is slightly different. More specifically, although PA11 droplets are present, a significant proportion of the particles are ellipsoidal in shape, and the distinction between the PLA and PA11 phases is much less visible.

The thermal behaviour was investigated using differential scanning calorimetry analysis. Table 2 summarises the results obtained, and Figures S3–S5 in the Supplementary Information show the heating and cooling scans. It appears that the presence of ZnO and *f*-ZnO does not affect the Tg of the PLA phase, suggesting that the PLA and PA11 phases remain almost separate. Conversely, the presence of both unmodified and modified ZnO particles increases both the Tm1 and Tm2 temperatures, suggesting an influence on the mobility of the biopolymer macromolecules. Overall, the degree of crystallinity of both the PLA and PA11 phases remains almost unchanged in the presence of ZnO and *f*-ZnO particles compared to the values obtained for the PLA/PA11 blend.

The photo-oxidation resistance of PLA/PA11 (70/30 *w/w*) and of materials containing 0.5, 1 and 2 wt.% of ZnO and *f*-ZnO was evaluated. The thin films were subjected to UVB exposure for up to 480 h, and the progress of photo-degradation was monitored by FTIR analysis at different exposure times. Typically, the thermo- and photo-degradation of blends, particularly biopolymer blends, occurs more rapidly than the degradation of individual components, considering the cumulative radicals that originate from each constituent. However, if the reinforcement particles significantly modify the morphology, for example, from droplet to co-continuous, the rate of degradation could be slowed down [35].

**Figure 6:**
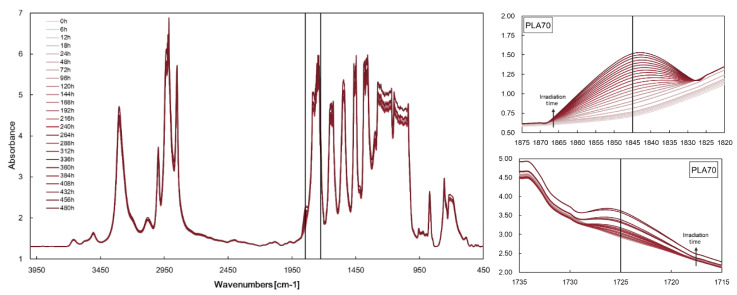
FTIR spectra (**left**) of PLA/PA11 = 70/30 *w/w* at different exposure times and selected zones in FTIR spectra (**right**), e.g., height of peaks at 1845 cm^−1^ and 1725 cm^−1^ at different exposure times.

However, the progress of photo-oxidation of PLA/PA11 blends can be profitably monitored following both the accumulation of anhydride functions (peak at 1845 cm^−1^), due to the PLA photo-oxidation, and the accumulation of carbonyl functions (peak at 1725 cm^−1^), due to the PA11 oxidation. Figure 6 shows the FTIR spectra at different exposure times (left) and selected zones in FTIR spectra (right), e.g., height of peaks at 1845 cm^−1^ and 1725 cm^−1^ at different exposure times. Both heights of peaks at 1845 cm^−1^ and 1725 cm^−1^ increase with increasing UBV exposure time.

**Figure 7:**
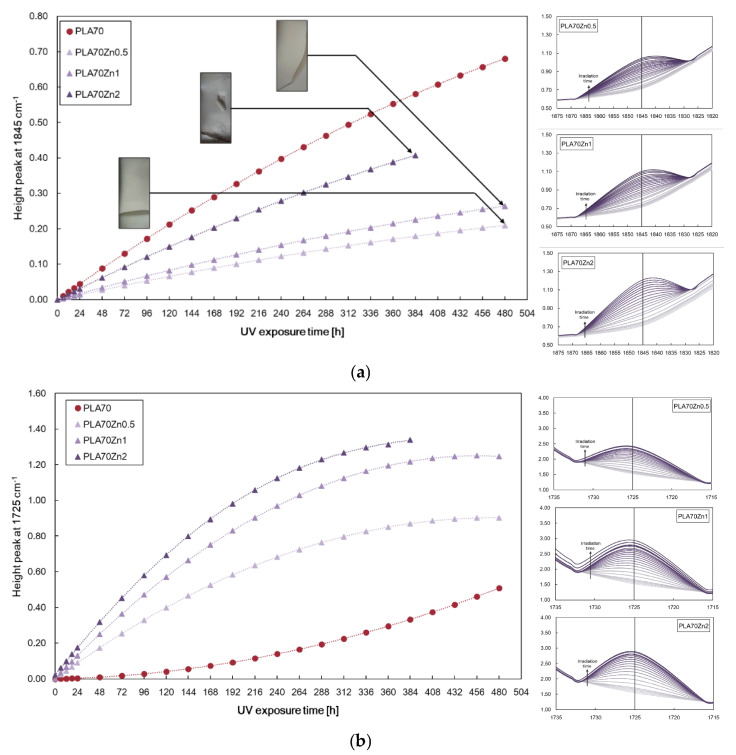
Height of peaks at (**a**) 1845 cm^−1^ and (**b**) 1725 cm^−1^ for PLA/PA11 = 70/30 *w/w* and films containing 0.5, 1, and 2 wt.% ZnO at different exposure times.

**Figure 8:**
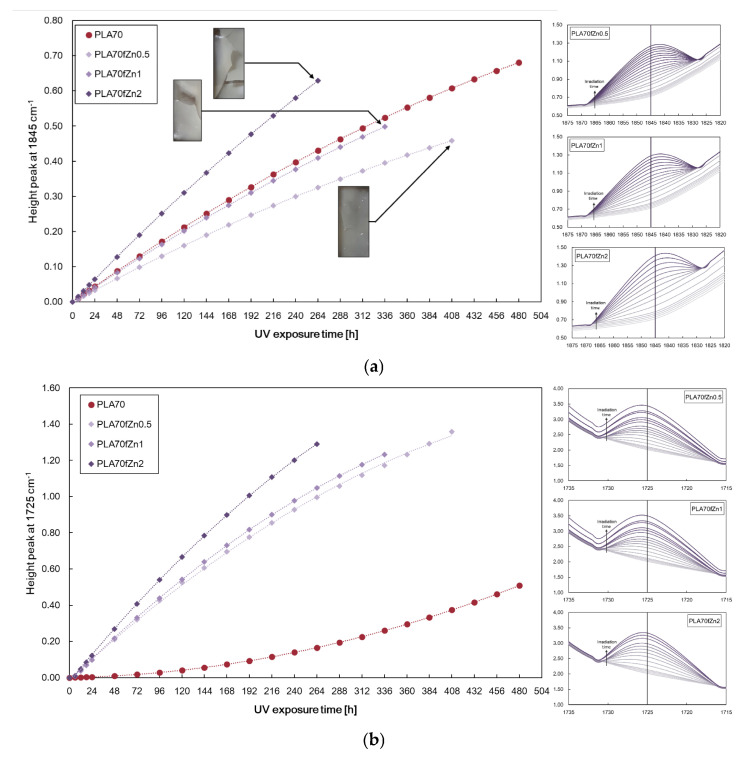
Height of peaks at (**a**) 1845 cm^−1^ and (**b**) 1725 cm^−1^ for PLA/PA11 = 70/30 *w/w* and films containing 0.5, 1, and 2 wt.% *f*-ZnO at different exposure times.

Figure 7a,b display the heights of peaks at 1845 cm^−1^ and 1725 cm^−1^ for PLA/PA11 = 70/30 *w/w* and films containing 0.5, 1, and 2 wt.% ZnO at different exposure times, and similarly, Figure 8a,b show the accumulations for the films containing 0.5, 1, and 2 wt.% *f*-ZnO.

The obtained results highlight that the presence of unmodified ZnO particles slows down the formation of anhydride functions in PLA (see Figure 7a) and promotes the formation of carbonyl functionalities in PA11 (see Figure 7b). These effects increase with increasing ZnO particle concentration. Interestingly, the presence of f-ZnO clearly accelerates the accumulation of carbonyl functionalities (see Figure 8b), while at high concentrations (i.e., 2 wt.% *f*-ZnO) it promotes the formation of anhydride functionalities (see Figure 8a). These results can be explained by considering that the stearic acid modifier promotes the formation of larger particles, which have a pro-degradant effect. The presence of the stearic acid modifier in the ZnO particles could favour the formation of free volume due to its miscibility with both biopolymeric phases, leading to a more complex system with reduced photo-oxidative stability.

### 3.2. Characterisations of Biopolymer Blend Containing s-TiO_2_

**Figure 9:**
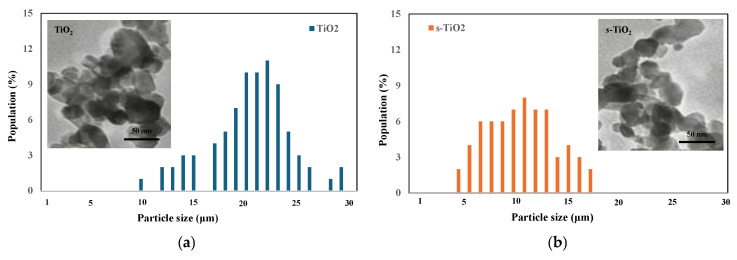
Count of particle size distributions determined by TEM observations of (**a**) TiO_2_ and (**b**) *s*-TiO_2_.

To investigate the effect of sonication on the particle size of TiO_2_, the particle size dimensions of both untreated and sonicated TiO_2_ (s-TiO_2_) were estimated by analysing TEM images. It appears that TiO_2_ exhibits the largest particle population at 20–22 µm, which is supported by the literature [36]. After sonication, this population is approximately half the size, i.e., around 10–12 µm, indicating the formation of smaller particles, as shown in Figure 9b.

Given the photocatalytic properties of TiO_2_ and its relatively high cost, it has been added to the PLA/PA11 blend at a concentration of 1 wt.%. Furthermore, sonication has been used as a sustainable and low-cost treatment to reduce particle dimensions and prevent aggregation during melt mixing.

**Figure 10:**
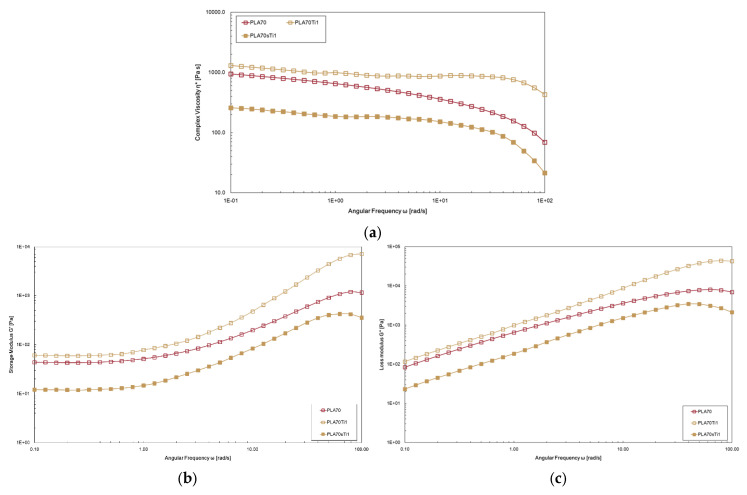
(**a**) Complex viscosity, (**b**) storage moduli, G′, and (**c**) G″ moduli trends of PLA/PA11 = 70/30 *w/w* (named “PLA70” in the figure) and materials containing TiO_2_ and *s*-TiO_2_ at 1 wt.%.

Figure 10a–c show the trends in the complex viscosity and G′ and G″ moduli of PLA/PA11 = 70/30 *w/w* (named “PLA70” in the figure) and of materials containing 1 wt.% of TiO_2_ and *s*-TiO_2_. The particles of TiO_2_ and *s*-TiO_2_ exert opposite effects on rheological behaviour. More specifically, TiO_2_ particles increase viscosity and moduli, exhibiting a reinforcing effect in the melt state, while *s*-TiO_2_ particles significantly decrease viscosity and moduli, exhibiting a plasticising effect in the melt state.

**Figure 11:**
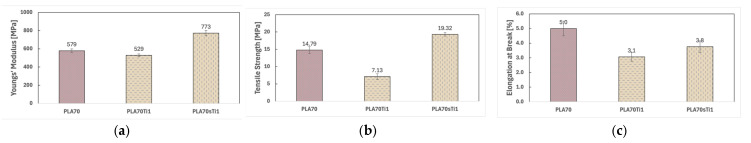
Main mechanical properties: (**a**) Young’s modulus, E, (**b**) Tensile strength, TS, and (**c**) Elongation at break, EB, of PLA/PA11 = 70/30 *w/w* (named “PLA70” in the figure) and materials containing TiO_2_ and *s*-TiO_2_ at 1 wt.%.

Therefore, even in the solid state TiO_2_ and *s*-TiO_2_ particles have different effects. More specifically, TiO_2_ at 1 wt.% causes a decrease in Young’s modulus (−8.6%) and tensile strength (−51.7%), whereas *s*-TiO_2_ particles increase both Young’s modulus (+25%) and tensile strength (+30%) compared to the biopolymer blend (PLA/PA11) values. Both composite materials are as fragile as the biopolymer matrix (EB < 5%), see Figure 11a–c.

**Figure 12:**
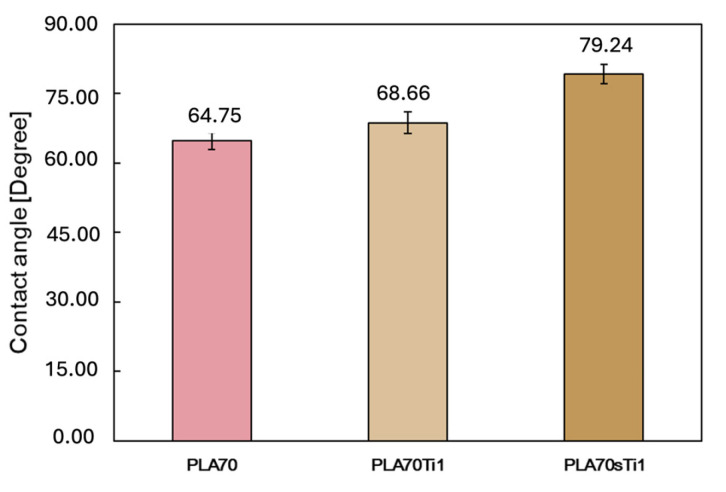
Water contact angle (WCA) measurements of PLA/PA11 = 70/30 *w/w* (named “PLA70” in the figure) and materials containing TiO_2_ and *s*-TiO_2_ at 1 wt.%.

The surface hydrophobicity of films containing 1 wt.% TiO_2_ and *s*-TiO_2_ was measured and the values obtained were compared to those of the matrix (see Figure 12). The presence of TiO_2_ increases the WCA value by around 6%, whereas the presence of *s*-TiO_2_ causes a significant increase in the WCA value by around 22%, suggesting a pronounced hydrophobic effect due to the presence of the particles on the film surface.

**Figure 13:**
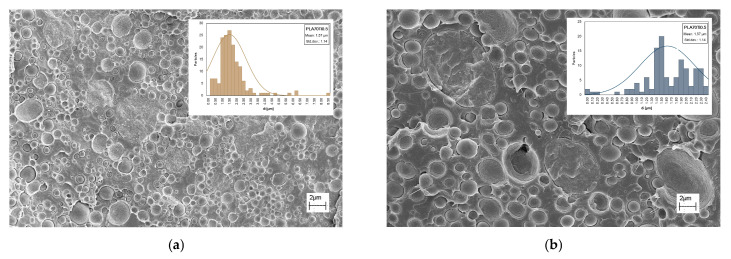
SEM observations and the inserts show the distribution of PA11 particle dimensions of PLA/PA11 = 70/30 *w/w* containing (**a**) TiO_2_ and (**b**) *s*-TiO_2_ at 1 wt.%.

The SEM observations have been performed, and the distributions of the PA11 particles’ dimensions have been calculated, see Figure 13 and Table 3. The dimensions of PA11 particles appear higher for the blends containing both TiO_2_ and *s*-TiO_2_, in comparison to the blend without metal oxide. This can be understood by considering that the untreated and treated TiO_2_ particles are allocated randomly, and both TiO_2_ and *s*-TiO_2_ do not prevent the formation of droplet morphology.

The heating and cooling scans of the PLA/PA11 = 70/30 *w/w* with added unmodified and modified TiO_2_ particles are shown in Figures S6 and S7 in the Supplementary Information, and the DSC data are reported in Table 4. Regarding the thermal behaviour of these materials, the Tg value of the PLA phase is not affected by the presence of the metal oxide, while the T_m,1_ and T_m,2_ temperatures change is, suggesting that the mobility of the macromolecules is influenced by the presence of both TiO_2_ and *s*-TiO_2_. The changes in the calculated crystallinity appear negligible, as can be seen in Table 4.

**Figure 14:**
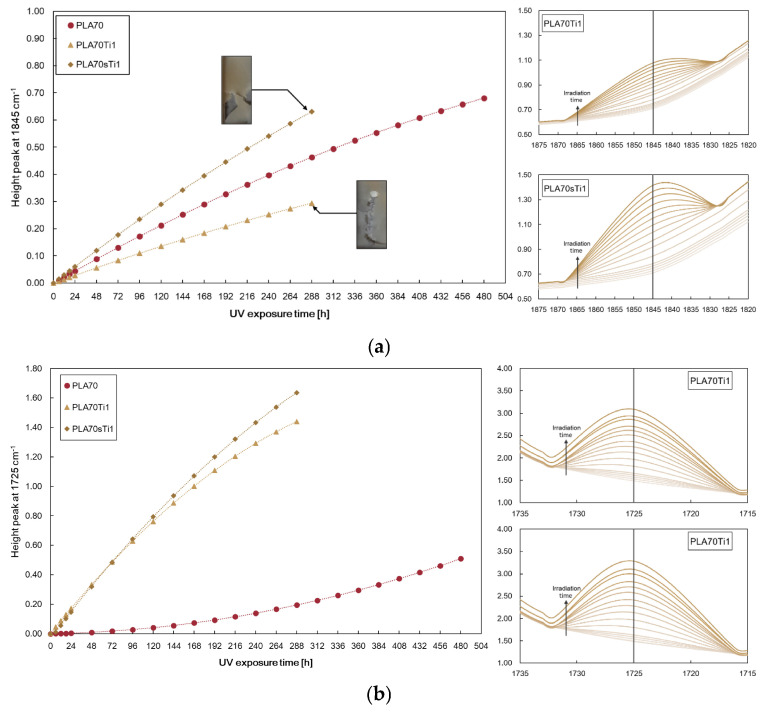
Height of peaks at (**a**) 1845 cm^−1^ and (**b**) 1725 cm^−1^ for PLA/PA11 = 70/30 *w/w* and films containing TiO_2_ and *s*-TiO_2_ at 1 wt.% at different exposure times.

The photo-oxidation study was carried out under the same conditions as those for the ZnO-containing samples. Figure 14a,b show the peak heights at 1845 cm^−1^ and 1725 cm^−1^ for the films containing both TiO_2_ and *s*-TiO_2_ compared to the blend matrix. The presence of TiO_2_ does not favour the accumulation of anhydride functions in the PLA phase upon UVB ageing: it only accelerates the formation of oxygen-containing groups in the PA11 phase. Interestingly, s-TiO_2_ exacerbated the accumulation of both anhydride and oxygen-containing groups in the PLA and PA11 phases, respectively. Therefore, *s*-TiO_2_ particles have a well-pronounced photocatalytic effect on the biopolymer blend.

## 4. Conclusions

This work is driven by the application of circular principles and the formulation of sustainable materials and focuses on the use of modified metal oxides as suitable additives for a biopolymer blend based on PLA and PA11. Specifically, stearic acid-modified ZnO (*f*-ZnO) and sonicated TiO_2_ (*s*-TiO_2_) have been introduced into a biopolymer blend via melt mixing. Spectroscopy and thermogravimetry analyses have estimated the presence of stearic acid tails on ZnO at approximately 4%. Sonication of TiO_2_ leads to the formation of particles with dimensions half the original size, as estimated by microscopy observations. The results obtained suggest that unmodified ZnO particles reinforce the PLA/PA11 blend in the melt state, whereas *f*-ZnO can plasticise the blend, reducing its viscosity. In the solid state, both ZnO and *f*-ZnO reinforce the blend, and this is more pronounced with increasing particle amounts. In terms of UVB resistance, *f*-ZnO particles, particularly at high concentrations, increase the number of oxygen-containing groups in the PA11 phase and the amount of anhydride functionalities in the PLA phase. In contrast, unmodified ZnO particles only affect the accumulation of oxygen-containing groups in the PA11 phase, with no negative effect on the PLA phase.

Interestingly, unmodified and sonicated TiO_2_ particles also have an opposite effect on rheological behaviour, as TiO_2_ reinforces the biopolymer blend while *s*-TiO_2_ reduces viscosity, exerting a plasticising effect. Furthermore, both TiO_2_ and *s*-TiO_2_ affect macromolecular mobility without influencing the PLA glass transition temperature. Once again, *s*-TiO_2_ particles clearly exert a photocatalytic effect on biopolymer blends, whereas unmodified TiO_2_ only promotes the formation of oxygen-containing species in the PA11 phase.

In summary, the proposed modification involving stearic acid-modified ZnO and sonicated TiO_2_ results in particles forming that do not alter the morphology of the droplet. The *f*-ZnO are probably mainly located at the interface between the biopolymeric phases or in the less-polar phase, whereas the *s*-TiO_2_ are randomly distributed with no identifiable preferential location. However, they negatively impact the materials’ photo-oxidation resistance, and this must be considered when designing biopolymer blends containing metal oxides for large-scale industrial applications, particularly modified ones.

## Figures and Tables

**Table 1:** Number of analysed particles (n_i_), average numerical diameter (*d_n_*) of PA11 particles in PLA/PA11 blend and blends containing ZnO and *f*-ZnO, and dispersion *D* = *d_v_*_/_*d_n_*.

Samples	Σni	$d_{n}\;[\mu m]$	D
PLA70	166	1.35 ± 0.11	1.41
PLA70 Zn0.5	204	1.02 ± 0.08	1.36
PLA70 *f*-Zn0.5	118	1.69 ± 0.14	1.13

**Table 2:** DSC data of neat PLA and PA11, blend PLA/PA11 = 70/30 *w/w*, and materials containing 0.5 wt.% ZnO and 0.5 wt.% *f*-ZnO.

Samples	Tg [°C]	Tc1 [°C]	Tc2 [°C]	Tm1 [°C]	Tm2 [°C]	Xc1 [%]	Xc2 [%]
PLA	62.23	96.18	-	174.54	-	14.59	-
PA11	-	-	146.27	190.10	-	-	
PLA70	61.78	97.04	147.16	167.76	183.29	8.92	2.71
PLA70 Zn0.5	61.73	97.66	147.23	174.11	189.38	10.90	3.56
PLA70 *f-*Zn0.5	61.51	97.98	148.62	173.67	188.82	8.68	2.27

**Table 3:** Number of analysed particles (n_i_), average numerical diameter (*d_n_*) of PA11 particles in PLA/PA11 blend and blends containing TiO_2_ and *s*-TiO_2_, and dispersion *D* = *d_v_*_/_*d_n_*.

Samples	Σn_i_	$d_{n}\;[\mu m]$	D
PLA70	166	1.35 ± 0.11	1.41
PLA70TiO_2_-1	132	1.57 ± 0.13	1.50
PLA70 s-TiO_2_-1	138	1.67 ± 0.18	1.55

**Table 4:** DSC data of blend PLA/PA11 = 70/30 *w/w* and materials containing TiO_2_ and s-TiO_2_ at 1 wt.%.

Samples	T_g_ [°C]	T_c,1_ [°C]	T_c,2_ [°C]	T_m,1_ [°C]	T_m,2_ [°C]	X_c,1_ [%]	X_c,2_ [%]
PLA70	61.78	97.04	147.16	167.76	183.29	8.92	2.71
PLA70 TiO_2_-1	61.79	96.3	152.46	173.49	188.33	10.43	1.01
PLA70 *s*-TiO_2_-1	61.29	92.33	150.64	173.38	188.36	9.01	2.26

## Data Availability

The original contributions presented in this study are included in the article/Supplementary Material. Further inquiries can be directed to the corresponding author.

## Supplementary Materials

The following supporting information can be downloaded at: https://www.mdpi.com/article/10.3390/polym17223000/s1, Supporting information containing: Figure S1. (a) G′ and (b) G″ trends of PLA/PA11/ZnO; Figure S2. (a) G′ and (b) G″ trends of PLA/PA11/f-ZnO; Figure S3. DSC traces of PLA/PA11 = 70/30 *w/w*; Figure S4. DSC traces of PLA/PA11 = 70/30 *w/w* containing 0.5 wt.% ZnO; Figure S5. DSC traces of PLA/PA11 = 70/30 *w/w* containing 0.5 wt.% f-ZnO; Figure S6. DSC traces of PLA/PA11 = 70/30 *w/w* containing 1 wt.% TiO_2_; Figure S7. DSC traces of PLA/PA11 70/30 *w/w* containing 1 wt.% s-TiO_2_.
