# Supplementary material for: Performance and Durability of Biopolymer Blends Containing Modified Metal Oxide Particles
Source: Polymers (Basel). 2025 Nov 11;17(22):3000. doi: 10.3390/polym17223000 (PMC12655913; doi:10.3390/polym17223000)
Supplement: Supplementary file 1 [file polymers-17-03000-s001.zip › polymers-3921580-supplementary.pdf]

Supplementary files

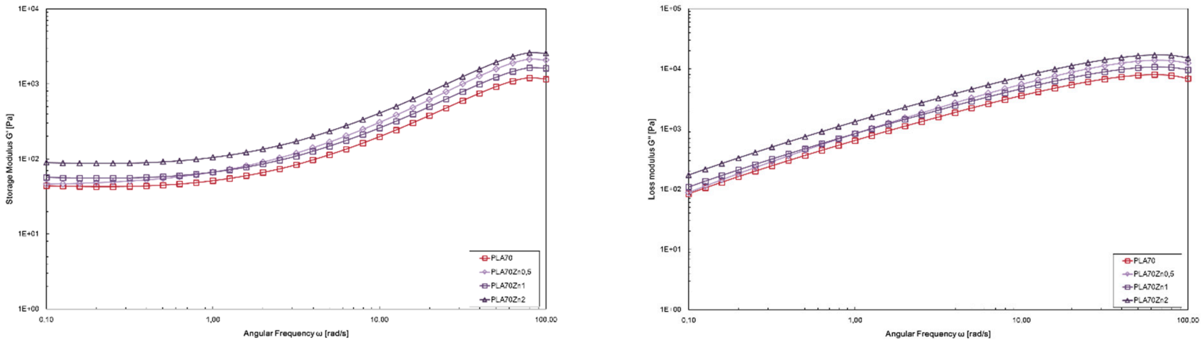

(a) **(b)**

Figure S1. (a) G’ and (b) G’’ trends of PLA/PA11/ZnO

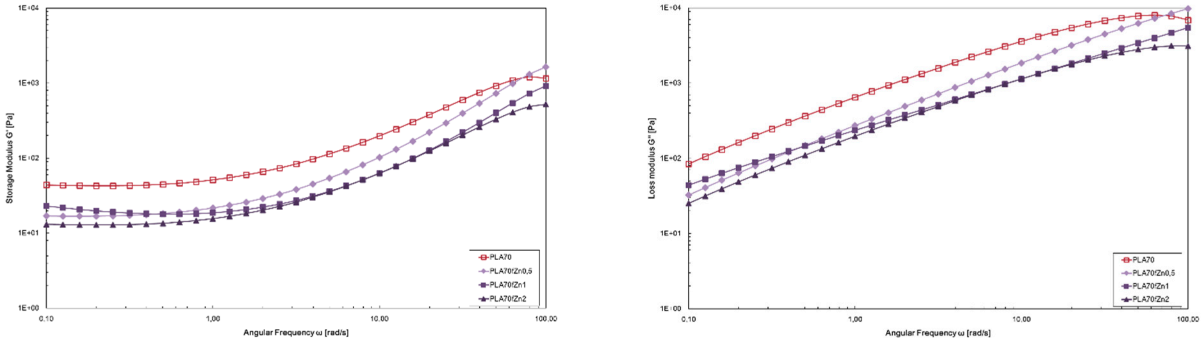

(a) **(b)**

Figure S2. (a) G’ and (b) G’’ trends of PLA/PA11/*f*-ZnO

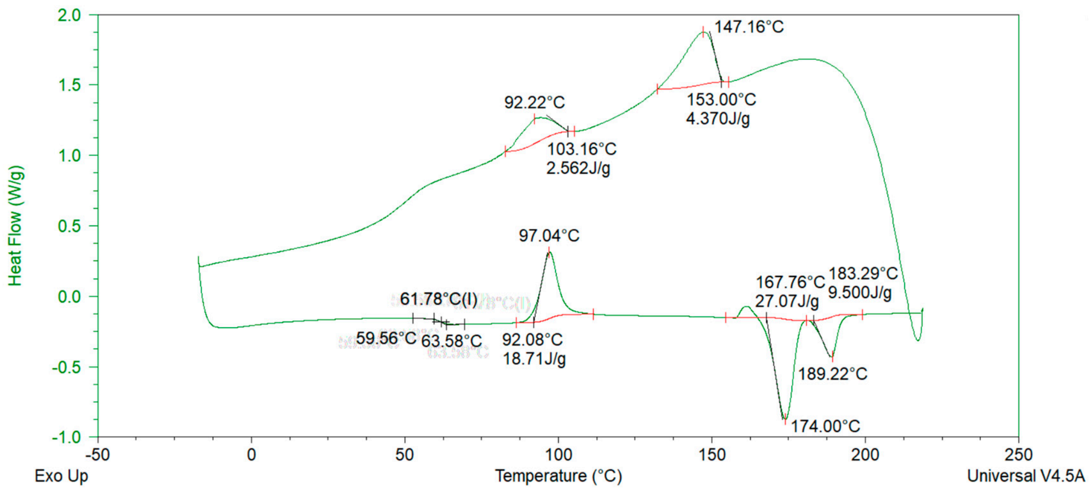

Figure S3. DSC traces of PLA/PA11 = 70/30 w/w

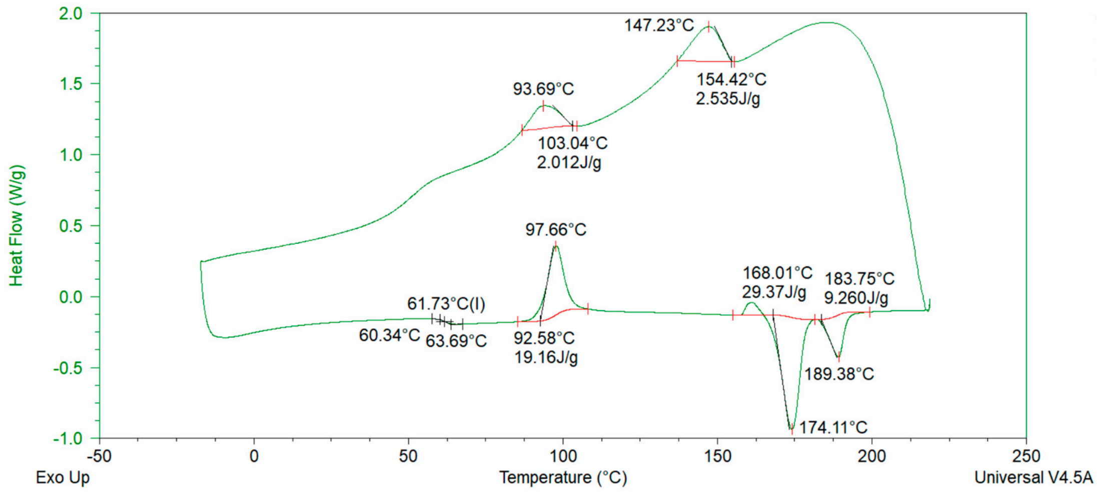

Figure S4. DSC traces of PLA/PA11 = 70/30 w/w containing 0.5 wt.% ZnO

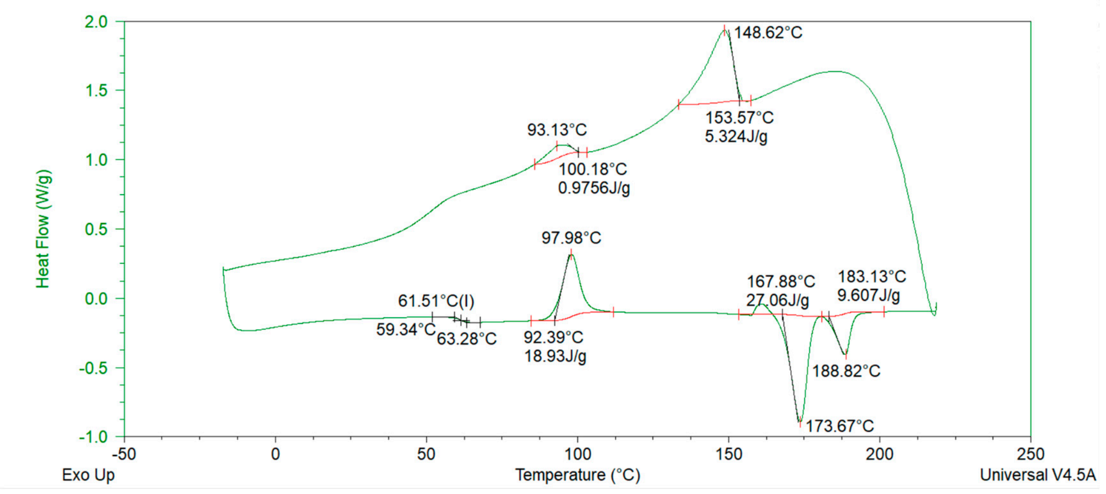

Figure S5. DSC traces of PLA/PA11 = 70/30 w/w containing 0.5 wt.% *f*-ZnO

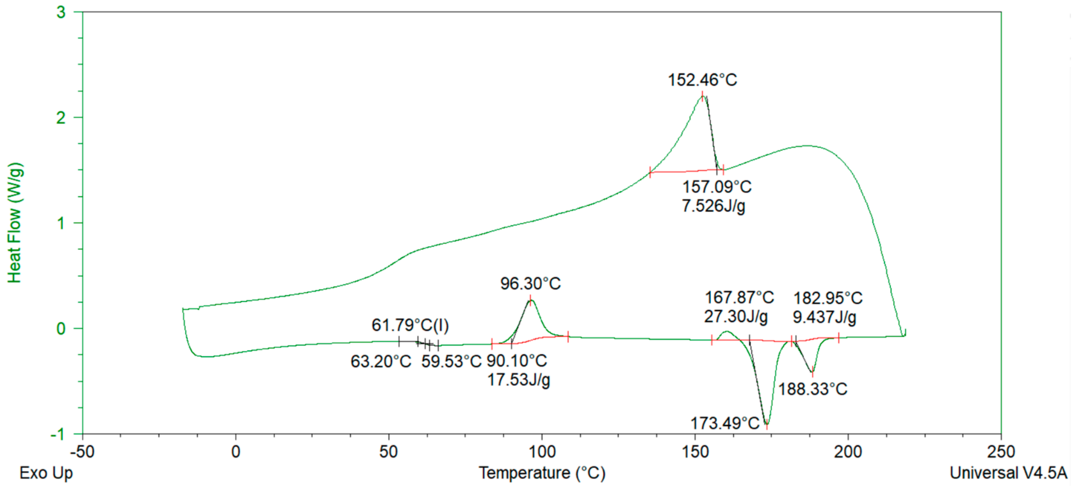

Figure S6. DSC traces of PLA/PA11 = 70/30 w/w containing 1 wt.%TiO_2

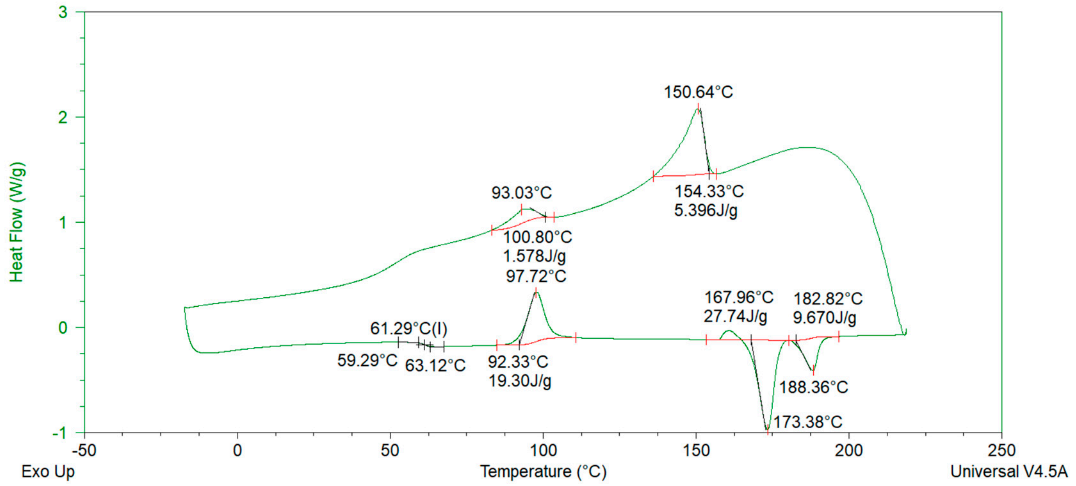

Figure S7. DSC traces of PLA/PA11 = 70/30 w/w containing 1 wt.% *s*-TiO_2
